# A case report of inflammatory pseudotumor of carotid artery with radiologic-pathological-surgical findings

**DOI:** 10.1186/s12872-015-0020-6

**Published:** 2015-04-09

**Authors:** Xudong Chen, Cheng Fei Zheng, Xiaoling Wang, Hannu Savolainen, Jin wei, Jianhui Li

**Affiliations:** Department of vascular surgery, the First Affiliated Hospital, Zhejiang University, College of Medicine, Hangzhou, Zhejiang 310003 China; Department of pathology, the First Affiliated Hospital, Zhejiang University, College of Medicine, Hangzhou, Zhejiang 310003 China; Department of Cardiovascular Surgery, University Hospital, Berne, 3010 Switzerland; Department of Hepatobiliary and Pancreatic Surgery, Key Laboratory of Organ Transplantation, Zhejiang Province, Key Laboratory of Combined Multi-organ Transplantation, Ministry of Public Health, First Affiliated Hospital, Medical College, Zhejiang University, Hangzhou, Zhejiang 310003 China

**Keywords:** Inflammatory pseudotumor, Carotid artery

## Abstract

**Background:**

Inflammatory pseudotumor is an unusual benign entity that can mimic malignancy in many different organ systems.

**Case presentation:**

We present a patient with a neck mass, which clinically and radiologically appeared to be a malignant tumor that encircled a segment of the left common carotid artery. The operation was performed and further histopathological evaluation confirmed this mass to be an inflammatory pseudotumor. After 4 years follow up, no recurrence was observed for this patient.

**Conclusions:**

Despite its rarity, IPT is important because it can simulate neoplastic disease. The contrast imaging may be helpful in differential diagnosis, but no findings are characteristic. The diagnosis of the IPT of carotid artery can be challenging for the surgeons. Certain diagnosis depends on either core biopsy or intra- or post-operative pathological examination. The surgical excision may be a curative option.

**Electronic supplementary material:**

The online version of this article (doi:10.1186/s12872-015-0020-6) contains supplementary material, which is available to authorized users.

## Background

Inflammatory pseudotumor is an unusual benign entity that can mimic malignancy in many different organ systems [1]. We describe a patient with a neck mass, which clinically and radiologically appeared to be malignant tumor. Further histopathological evaluation confirmed this mass to be an inflammatory pseudotumor. The authors ensured that the work described has been carried out in accordance with The Code of Ethics of the World Medical Association (Declaration of Helsinki) for experiments involving humans.

## Case presentation

**Figure 1:**
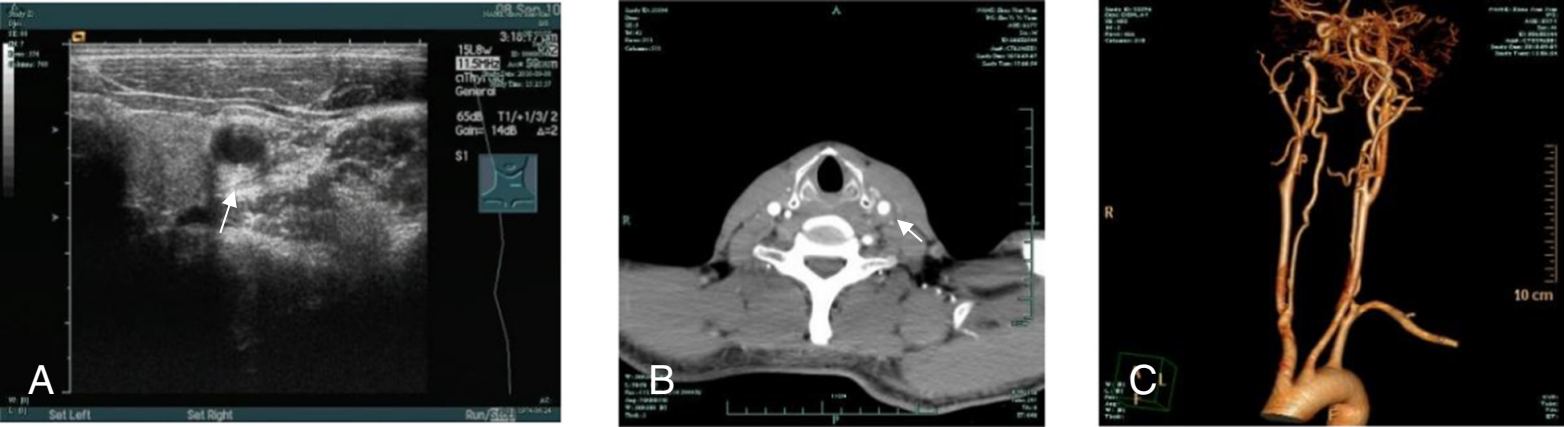
The BUS (**A**) and three-dimensional CT angiography (CTA) (**B**, **C**) revealed a mass wrapping around the segment of the vessel from left common carotid artery to internal carotid in the left neck (carotid body tumor suspected).

**Figure 2:**
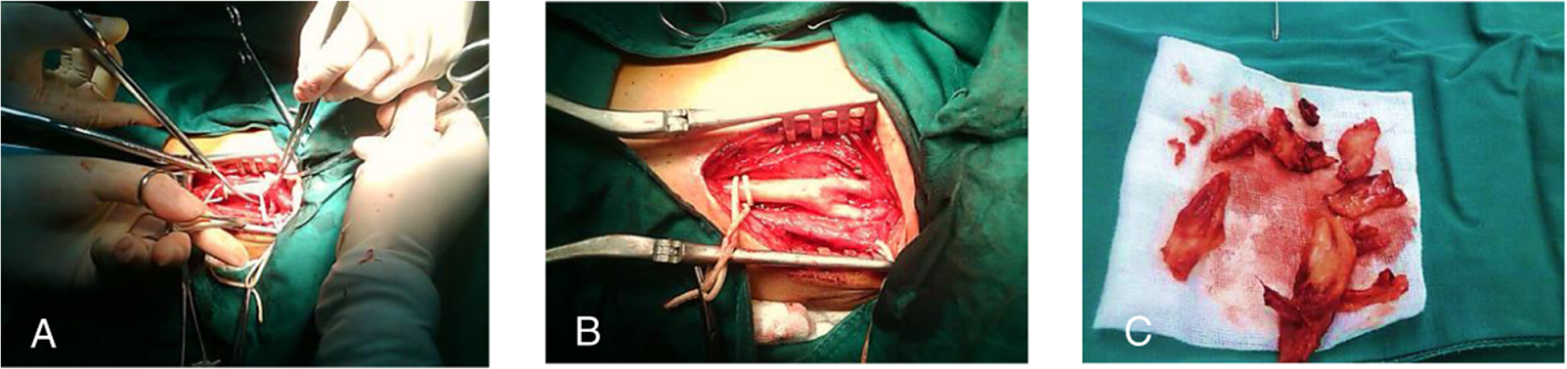
The surgical findings for the patient undergoing operation. During the operation, the tumor was located in the segment from left common carotid artery to internal carotid (**A**), and after the resection of the tumor, the vessels were clearly reserved (**B**), the cut view of the tumor post operation (**C**).

A 37-year-old man having a neck mass with a history of one month of headache, nausea, and dizziness was considered. Five days prior to hospitalization, the patient had an irritating cough, hoarseness of voice, chest tightness and shortness of breath. No cervical lymphadenopathy was palpated. B type ultrasound (BUS) and Computed Tomography (CT) demonstrated a mass encircling a segment of vessel from the left common carotid to the internal carotid artery in the left neck (Figure 1). Therefore, a carotid body tumor was suspected. Chest radiography showed no pathology. Standard tumor markers were negative. Other routine laboratory examinations, including HIV and syphilis testing, were within normal limits. Physical examination showed conjunctival hyperemia, which can be due to the compression of the ipsilateral jugular vein by a 2 cm palpable soft mass in the left neck. The patient had no history of neck infections or tobacco or alcohol consumption. His past medical and surgical history was uneventful. An extensive resection was performed and a 3-cm well-demarcated lesion within the carotid sheath revealed (Figure 2), the tumor located in the segment from left common carotid artery to internal carotid. The tumor can be dissected from the carotid arterial adventitia, and the vessels were clearly reserved without reconstruction.

**Figure 3:**
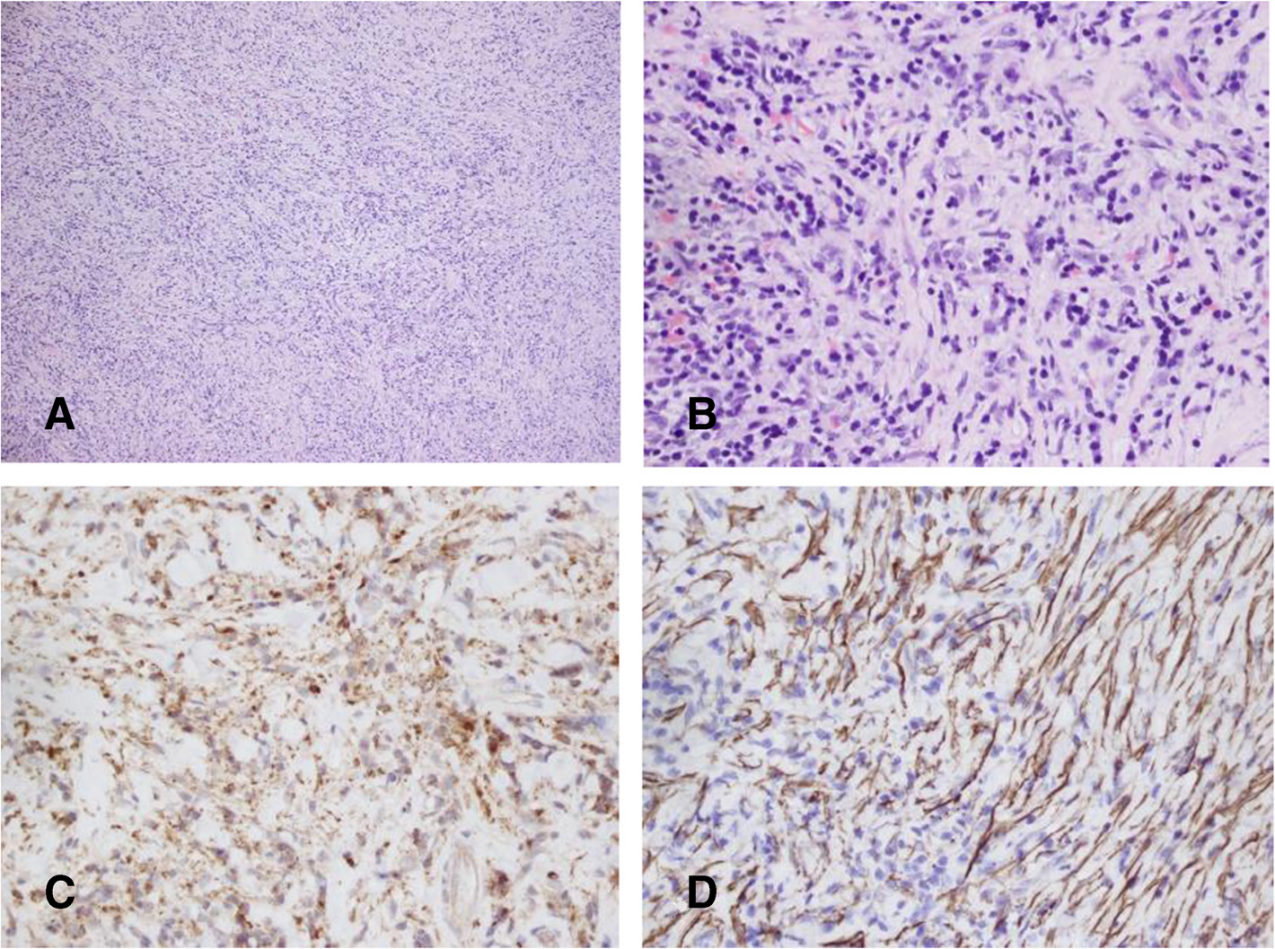
Microscopic examination revealing dense mixed infiltrate of lymphocytes, plasma cells, and histiocytes, eosinophils ,among a background of collagenous fibrils, (Hematoxylin and eosin staining, **A**: original magnification × 100, **B**: original magnification × 400). Immunohistochemical staining showed positive reaction to CD68 (**C**), Smooth Muscle Actin (SMA) (**D**); (Original magnification × 400). These histological findings were consistent with a diagnosis of carotid artery IPT.

The histopathologic examination showed that the lesion was made up of heterogeneous inflammatory cells with fibrotic proliferation (Figure 3). Several enlarged lymph nodes were identified; the largest one was 0.8 cm in size. Recovery was uneventful. No steroid treatment was given after the operation. The patient was followed up with no evidence of recurrence up to now.

## Discussion

Inflammatory pseudotumor(IPT), as a benign chronic inflammatory mass with the proclivity to imitate a malignant process [1] was initially described by Brunn in 1939 [2]. Since then, various synonyms such as inflammatory pseudotumor (IPT), inflammatory myofibroblastic tumor (IMT) or plasma cell granulomas have been used to describe this inflammatory solid tumor containing spindle cells, myofibroblasts, plasma cells and histiocytes [3]. Up to now, IPT has been reported to involve a variety of organs and tissues in the body. The most common presentations are those in the head, neck and the orbit. At times, it is seen in the lungs, but carotid artery IP is very rare [4]. To our knowledge, 8 cases of carotid artery related pseudotumor, including the current case we described have been reported; the age ranging from 40 years old and above. Out of the eight cases, 4 were female. All the instances are solitary, and one side involved. Three were asymptomatic, and the remaining individuals manifested constitutional symptoms including headache, nausea, facial edema, dizziness, and swallowing difficulty. In other locations, these associated symptoms and signs of IPT were resolved once the mass was excised or treated with steroid [4-10] (Additional file 1: Table S1).

We report here a case of inflammatory pseudotumor located at the carotid artery. Imaging studies showed a mass encircling a segment from the left common carotid to the internal carotid artery in the neck raising suspicion of a carotid body tumor. To avoid the risk of damage to the carotid artery and to relieve symptoms caused by compression, a biopsy was omitted, and a wide surgical excision was performed directly. Although the clinical presentation mimicked a malignant tumor, no evidence of malignancy was seen at operation. An intraoperative frozen section biopsy showed an inflammatory process composed of a mixture of lymphoplasmocytic infiltrate, histiocytic and spindled fibroblastic cells. Further pathological studies confirmed the lesion to be mainly composed of mixed T and B lymphocytes, and histiocytes. Additionally, reactive changes in the local lymph nodes support the role of an inflammatory process in the formation of the mass.

The etiology and pathogenesis of IPT are obscure. Potential mechanisms include an inflammatory reaction secondary to trauma, an autoimmune reaction or infection. The most likely cause is infection. In our case, immunohistochemical staining was done to exclude paraganglioma or another malignancy. It indicated SMA (+) and CD68 (+) possibly correlated with generation of contractile force of fibroblasts and inflammatory formation. The staining was negative for anaplastic lymphoma kinase and follicular dendritic cell markers CD21 and CD35. CD3, CD20, CD163, CD30, CD34, Syn, CGA, S100.

Despite its rarity, IPT is important because it can simulate neoplastic disease. However, its diagnosis can be challenging. The clinical behavior of inflammatory pseudotumor is similar to that of a neoplasm. For a carotid lesion, differential diagnosis includes carotid body tumor (also known as chemodectoma or paraganglioma) [11], fibro-muscular dysplasia [12], Wegener’s granulomatosis [13], extramedullary plasmacytoma [5] and carotid artery pseudoaneurysm.

All these lesions can present with cough, headache, nausea, dizziness and other non-specific symptoms. In case the clinical presentation and imaging studies show a suspicious appearance, it is often initially diagnosed as a malignant tumor. Contrast imaging (CT) and Magnetic resonance imaging (MRI)) may be helpful in the differential diagnosis, but no findings are characteristic. Three-dimensional reconstruction (3-D) of the lesion can be used to define its anatomy, and it may help the surgeon to plan the operation. Certain diagnosis depends on either core biopsy or intra- or post operative pathological examination [8].

Standard treatment of the inflammatory pseudotumor of carotid artery is not yet established. However, it can simulate a malignant process. Local expansion may warrant prompt surgical treatment. Surgical excision also appears to be curative. The extent of resection depends on preoperative imaging and intraoperative findings. The surgeon must be cautious when performing radical surgery to avoid damaging blood vessels or nerves. Intraoperative examination of a fast frozen section can be diagnostic [4,6]. Corticosteroid treatment [5,7,14] and radiotherapy [15] may be useful alternatives in those patients with unresectable masses [10], but a definitive diagnosis often requires a surgical biopsy [7].

## Conclusion

In conclusion, we presented a case of inflammatory pseudotumor arising from carotid artery. IPT is very rare but may simulate a malignancy and should be remembered as an option in a patient presenting with a neck mass. The diagnosis and management can be very challenging. Findings on imaging may not be characteristic but are helpful to guide the surgeon. Certain diagnosis depends on histopathological examination. Surgical excision appears to be curative.

## Consent

Written informed consent was obtained from the patient for publication of this Case report and any accompanying images. A copy of the written consent is available for review by the Editor of this journal.

## Additional file

Additional file 1: Table S1. Inflammatory pseudotumor (IPT) related to the carotid artery.
